# Revealing the missing links in the chain of relevant information from the clinicians to pathologists

**DOI:** 10.12669/pjms.41.7.11928

**Published:** 2025-07

**Authors:** Abbas Ghaffari, Shagufta Nasir Pervez, Zia ur Rehman, Bakhtawar Kamal

**Affiliations:** 1 Abbas Ghaffari Resident Medical Officer, Department of Histopathology, Hayatabad Medical Complex, Peshawar, Pakistan; 2 Shagufta Nasir Pervez Associate Professor, Department of Histopathology, Hayatabad Medical Complex, Peshawar, Pakistan; 3 Zia ur Rehman Resident Medical Officer, Department of Histopathology, Hayatabad Medical Complex, Peshawar, Pakistan; 4 Bakhtawar Kamal Resident Medical Officer, Department of Histopathology, Hayatabad Medical Complex, Peshawar, Pakistan

**Keywords:** Biopsy request, Documentation practices, Information Adequacy, Pathologist, Requisition forms, Scarcity of clinical information

## Abstract

**Objective:**

To evaluate the adequacy of demographic, clinical, and specimen-related information provided to the histopathology laboratory.

**Method:**

Total 400 biopsy requisition forms were studied retrospectively for the presence and absence of 20 different variables. This cross-sectional and single-centric study was conducted in a tertiary care setting in Peshawar Pakistan in year 2024. Data was collected in Microsoft Excel and analyzed with SPSS v.22. Frequencies, percentages, and mean percentage of the documented variables were determined. Scoring the documented variable as one and the undocumented variable as zero, RFs were scored out of a total score of 20 and mean score was calculated.

**Results:**

Only the name was written on 100% of forms, followed by MRN (95.5%), and the investigation required (91.25%). However, only 36% and 35.75% of forms contained the patient’s age and gender respectively. Essential information like clinical history, specimen nature, and site of biopsy were provided on 82.25%, 27.5%, and 50.25% of RFs respectively. Names of the requesting authorities appeared only on 24% forms. No form contained all the 20 variables. The mean percentage of all the documented variables came out to be 39.76%. On scoring, the minimum score was three while the maximum score was 15. The mean and standard deviation of the scores was 7.95±2.74.

**Conclusion:**

Doctors provided the patient identifiers adequately but clinical history and specimen-related data were not adequate. Also, the requesting doctors did not provide their own information on the RFs.

## INTRODUCTION

Biopsy is the gold standard for diagnosing lesions at the tissue level. A biopsy requisition form (RF) is the first communication between the requesting doctor and a pathologist. Ideally, for a swift and accurate definitive diagnosis in fewer resources, the provision of adequate demographic, clinical and specimen-related information to the pathologist is necessary. On the contrary, lack of the said information is a matter of great concern for pathologists around the globe, for it may increase the chances of wrong patient identification, erroneous diagnosis, false reporting, increased turnaround time (TAT),^1^,^2^ human errors,^3^ increased human efforts,^2^ and increased resource consumption.^2^,^4^,^5^

In our country Pakistan, only a few studies have been published that provide insight into whether the information of the patient provided to pathologists by the clinicians is sufficient or not and how this lack of information affects the diagnosis and reporting in pathology. However, these studies do not give a comprehensive overview of all the needed information to the pathologists. Therefore, studies to quantify the adequacy of demographic and clinical data provided to the pathologists are much needed in Pakistan. Our study provides the relevant statistical data on this subject however studies about its resultant errors and other implications are still much needed.

Our study provide the relevant and comprehensive statistical data on the subject. The results were compared to those of neighboring countries and the United Kingdom. Also, in our study, we have discussed the importance of all the necessary information needed to the pathologists that would, otherwise, be look irrelevant to a maximum number of doctors requesting a biopsy. Moreover, many possible reasons behind the scarcity of the said data and its possible impact on the diagnosis-making process, in the light of already available literature, are discussed in this article.

## METHODS

This retrospective single-centric study was conducted to quantify the demographic, clinical and specimen-related data provided to the pathologist and to check its adequacy at Histopathology laboratory in a tertiary care hospital in Peshawar in year 2024.

### Sample size:

The sample size was calculated using the online WHO calculator 1.1, with a population proportion of 0.5, a confidence interval of 0.95, and a margin of error 0.05. The calculated sample size for the study was 385 RFs which is rounded off to 400 RFs.

### Inclusion criteria:

- RFs sent for tissue biopsies in the year 2024.

### Exclusion criteria:

- RFs for trephine biopsies and cytology were excluded as their RFs are different from the biopsy RF.
- Biopsy RFs received before the first of January 2024 and after 31^st^ December 2024.

### Sampling Technique:

Four hundred RFs sent from different wards, were selected with convenient non-probability sampling technique.

### Data collection:

Biopsy RFs were retrieved within the laboratory and studied for the presence of 20 variables comprising of name, age, gender, address, contact number of the patient, ward, bed number, medical record (MR) number, clinical history, presumptive clinical diagnosis, procedure, intra-operative findings, radiological findings, specimen nature, site of biopsy, the date on which biopsy is taken, ordering surgeon/physician’s name, sign, contact number, and the investigation required. On each RF, the presence of the above-mentioned variables was studied.

### Steps of measurement of variables:

The data was collected directly into the Microsoft Excel. Even a bit of information was considered and categorized as “Yes” if provided, and “No” if not provided. Where a doctor used his stamp, it is considered as information about his name, ward and signature. If a variable was present on the RF, it was given a score of one and a score of zero if absent. Each RF got a particular score.

### Data analysis:

The compiled data was then analyzed with SPSS version 22. The adequacy of information regarding each variable was determined in terms of frequency and percentages. The adequacy of the overall information provided to the pathologist was determined by calculating the mean of the percentages of the individual variables. Frequency, percentage and a mean of the cumulative scores were calculated, which showed how many and how much, individual RFs were filled.

### Ethical approval:

The study was approved by the Ethical Review Committee/IREB on 6^th^ January 2025 under the reference number 2372.

## RESULTS

Out of 2301 biopsy requests received in the year 2024, we selected 400 RFs (n=400). Among these 400 RFs, 278 RFs (69.5%) were sent from Gynaecology and Obstetrics, while 118 RFs (29.5%) were sent from other General Surgery and Allied wards i.e. Plastic Surgery, Neurosurgery, ENT, Cardiac Surgery, Urology, Thoracic Surgery and Orthopedics wards. Four RFs (1%) were sent from Medical & Allied wards i.e. Medicine, Pediatrics and Oncology wards. Only name was the variable that was written on all (100%) of RFs, followed by MRN and the investigation required, with frequencies of 382 (95.5%), and 365 (91.25%) respectively. Clinical history, was present only on 329 (82.25%) RFs while only 92 (23%) RFs had the presumptive diagnosis. Only 9 (2.25%) RFs had radiological findings written on the RF (Table-I). There was not a single RF that had all the required data documented. When mean of the percentages of all variables were calculated, it came out to be 39.76%. Moreover, each variable, if present on an individual RF was scored as one and if absent, scored as zero, giving a collective score out of a total score of 20 for each individual RF (Table-II). A score of six was the most common which has a frequency of 64 (16%). The minimum score was three and the maximum was fifteen. Not a single RF scored 20 out of 20. The mean of this cumulative score was calculated, and it came out to be 7.95±2.74.

**Table-I:** Frequencies and percentages of the present and absent variables on RFs. (n=400).

Variable	Yes	No	Yes%	No%
Name	400	0	100	0
MR NO	382	18	95.5	4.5
Age	144	256	36	64
Gender	143	257	35.75	64.25
Address	24	376	6	94
Patient contact	170	230	42.5	57.5
Ward	153	247	38.25	61.75
Bed No	52	348	13	87
Clinical History	329	71	82.25	17.75
Presumptive Diagnosis	92	308	23	77
Operative Findings	25	375	6.25	93.75
Radiological Findings	9	391	2.25	97.75
Procedure	244	156	61	39
Specimen Nature	110	290	27.5	72.5
Site of Biopsy	201	199	50.25	49.75
Date of Biopsy	110	290	27.5	72.5
Required Investigation	365	35	91.25	8.75
Ordering Surgeon	96	304	24	76
Surgeon contact	16	384	4	96
Doctor’s Sign	116	284	29	71

**Table-II:** Frequency and percentage of the scores of individual RFs. (n=400).

Score	3	4	5	6	7	8	9	10	11	12	13	14	15
Frequency	6	24	53	64	55	47	26	44	28	25	19	7	2
Percent	1.5	6	13.3	16	13.8	11.8	6.5	11	7	6.3	4.8	1.8	0.5

## DISCUSSION

Ideally, for a correct identification of the patient, there must be at least two identifiers on the RF as well as on the specimen container.^6^,^7^ Similarly the clinical history of the patient and findings regarding the specimen is the most essential data and should be provided on each and every RF. In our study, name was the only variable that was present on 100% of RFs, followed by the MRN (95.5%). Three hundred and fifty (87.5%) RFs contained both the name and MRN. In the rest of 50 RFs, 48 (12%) had another identifier like age, gender, address, or patient contact number. Only two RFs (0.5%) had only one identifier. Collectively, 99.5% of RFs were following the criteria. The required investigation, present on 365 (91.25%) RFs was the third most frequently provided data. Despite that mere a chief complaint was also considered as a clinical history, it was present only on 329 (82.25%) RFs and most of the times, inconclusive. The procedure done (61%), intraoperative findings (6.25%), radiological findings (2.25%) and presumptive diagnosis (23%) were also not up to the mark. Similarly, nature (27.5%), site (50.25%) and date of the specimen collection (27.5%) as well as the personal data of the ordering physician was inadequate in our study (Table-I).

A clinical audit conducted in Pakistan revealed that name, MRN and gender were stated on 99.4%, 64.6% and 93.7% of RFs respectively but the clinical history, operative findings and radiological findings were only available on 82.3%, 53.7% and 9.1% RFs respectively.^8^ In an another study conducted in Pakistan, age and gender were mentioned on 94% and 86% of RFs respectively but the clinical history was reported to be present on 64% RFs only.^9^ In a study conducted in India, these four variables viz name, age, gender, and IP/OP number (IP/OP: In-patient/out-patient number equivalent to MRN in our study) were stated on 100%, 98.3%, 98.3%, and 100% of RFs respectively while clinical history on 35% of RFs only.^10^ The counts are better in the study conducted by J.L. Burton and T.J. Stephenson.^11^ Fariba Abbasi et. al. reports the patient’s identifiers and demographic data like name, age and gender were written on 99.8%, 91.96%, and 72.5% of RFs respectively but clinical history on 80% RFs only. A more detailed comparison can be seen in Table-III. This comparison suggests that the clinicians were more compliant to provide at least two identifiers of the patient however the counts of clinical and specimen-related information were markedly below the benchmarks.

**Table-III:** Amount of the information provided on biopsy RFs in various studies.

Variable (%)	Our Study	J L Burton et al.	Fariba Abbasi et al.	Dr Priyadharisini et al.	Mishaal Shan Siddiqui et al.	Muhammad Ashraf Sharif et al.
Sample size	n=400	n=2000	n=2040	n=114	n=175	n=500
Name	100	100	99.8	100	99.4	NA
Medical Record No	95	99.8	NA	100	64.6	NA
Age	27	99.6	91.96	98.3	NA	94.2
Gender	31	NA	72.25	98.3	93.7	86
Address	5	93.1	NA	NA	NA	NA
Patient contact number	44	NA	93.8	NA	28	NA
Ward	35.6	NA	NA	85	97.7	NA
Bed No	10	NA	NA	NA	52.8	NA
Procedure	65	NA	NA	89.4	NA	NA
Specimen Nature	26	98.9	12.1	NA	74.9	NA
Site of Biopsy	43	NA	92.8	77	87	87
Date of Biopsy	25	98.2	NA	100	NA	NA
Clinical History	80.7	93.9	80	35	82.3	64
Differential Diagnosis	25	53.1	82.6	94.7	93.7	
Operative Findings	3	NA	NA	NA	53.7	NA
Radiological Findings	2	NA	5.2	Included in Previous Investigations	9.1	NA
Doctor Name	19.8	47.5	98.7	38.5	98.3	77
Surgeon contact	3	33.3	0.05	NA	19.4	
Doctor’s Sign	28	91.5	NA	100	NA	
Investigation Requested	98	97.4	NA	NA	NA	NA
Previous Investigations	NA	NA	12.5	30.7	18.9	NA

Deficient and inadequate information may contribute to erroneous biopsy reports. A study conducted in the United States shows that among 60043 deficient RFs, wrong specimen identifiers contributed to 9.6% of errors while discrepant or missing information was implicated in 77% of errors.^12^ A study in Thailand, finds 26.81% cases of erroneous diagnosis due to wrong patient identification.^13^ Another study in Portugal reveals that in a sample of 10574 cases, 330 cases (3.1%) were found with errors. Among these errors, 65% were related to RFs, i.e. absence of RFs (10.6%), patient identifiers (1.2%), clinical information (15.8%), sample identification (35.1%), and clinician identification (2.1%).^14^ Siddiqui MS et al. reports that among the inconclusive biopsy reports, 78% had incompletely filled RFs.^8^ A famous study titled as “Clinicians Are From Mars and Pathologists Are From Venus” reveals that clinicians usually misinterpret such reports.^15^

The potential reason behind the adequacy of patient’s identifier’s data and that of investigation required may be attributed to the software used in the hospital i.e. HMIS (Hospital Management Information System), the minimum requirements for which is the name, MRN and the investigation to be ordered. Development and improvement in such software may improve clinical information delivery, and reduce the risks of errors and turn-around time.^16^,^17^ Also the identifiers are the least information required in a laboratory and the receptionist ensure its availability more religiously.

The inadequate clinical and specimen related information may be due to the increased workload on the doctors.^18^ It also puts a question mark on the concept of the doctors about the biopsies and their requirements. Non-availability of the specified biopsy RFs may also be a potential reason.^19^ Moreover, sometimes, the clinicians keep the pathologist blind intentionally, ignoring the fact that this blindness may affect the precision of definitive diagnosis.

In the absence of a clear clinical context, decision-making among many potential diagnoses such as benign versus malignant tumors or reactive versus neoplastic lesions, becomes increasingly difficult. For instance, a granuloma in a tissue may be of infective or inflammatory origin, may be due to a foreign body, due to a certain drug, or may be in a setting of neoplasia.^20^-^22^ The radiological findings are specially much needed in neural tissue biopsies,^23^ and bone biopsies.^24^ Specimen nature and description of the laterality are both necessary for reporting on the margins of the tumor and where tumor grading is required because, in comparison to excisional biopsy, there are high chances that high grade tumor cells may be missed in Fine Needle Aspiration or Punch biopsies. The site of the lesion is important where the tissues have the similar surface epithelium or where the tissue architecture is distorted or making the right choice of Immuno-Histo-Chemical (IHC) marker to be applied especially when one try to decide about the origin of tumor^25^ and type of tumor as some markers may come positive in many tumors. Without the information of the site of the biopsy the pathologist may start with a wide range of markers which itself carries the risk of false positives beside that the expenses and turnaround time increases.

Although many studies are published on this subject in the region, we think our study has some relative strengths given as following:

1. The sample size is adequate and was properly calculated using WHO calculator.
2. The methodology and data is reproducible.
3. A proper comparison was made with other similar studies published in the region and in a setting of standard health system like United Kingdom.
4. Each individual RF was scored and a mean score represented the amount of provided information on the majority of RFs.
5. Recommendations for improvement were given.

### Limitations:

Following are the limitations we faced in our study:

1. The study was a retrospective and single centric study. Future studies should be prospective to represent the real time situation. Also the practices may vary center to center.
2. The sampling technique is Convenient Non-probability sampling method.
3. Majority of the RFs were sent from Gynecology and Obstetrics and RFs from other departments are relatively smaller in number.
4. RFs were not specifically designed for biopsy requests. The RFs studied was general and for all type investigations, lacking a specific field for some of the variables.
5. Even the chief complaint of the patient was considered as a clinical history while an adequate clinical history contain many modalities.
6. The errors caused by inadequacy of clinical and specimen information could not be studied due to the retrospective design of the study and non-availability of the record of errors.
7. Statistically significant association of the said inadequate information with specific reasons could not be studied.

## CONCLUSION

The patient’s demographic, clinical and specimen-related information provided to the pathologists along with biopsy requests, was inadequate. Though the doctors seemed to be more compliant to provide patient identifiers, they did not provide enough clinical history, their findings and also their own information. This much scarcity of clinical and demographic information significantly contributes to many types of errors in the process of diagnosing and reporting.

The poor documentation practices can be improved by improving the concept of the clinicians about the biopsy requirements. The receptionist should be trained enough to ensure the availability of the said information. Awareness sessions should be arranged in different wards to encourage the requesting clinicians to provide adequate clinical history along with the specimen. Undergraduate medical students can be educated by teaching them the pre-analytical SOPs of specimen handling. Introducing Electronic Medical Records (EMR) software and specified biopsy RFs may also improve the poor documentation practices.

### Recommendations

Future studies may be aimed to determine the errors resulting from the inadequacy of relevant information. Also the results of the practical steps taken to improve the situation must be studied and shared with the medical fraternity.
